# Point-of-Care Ultrasound in the Diagnosis of Pott’s Puffy Tumor in a Pediatric Patient

**DOI:** 10.7759/cureus.86980

**Published:** 2025-06-29

**Authors:** Sania Shahid, Sreya Varanasi, Amer Salman, Thaer S Abohamra

**Affiliations:** 1 Pediatric Emergency Medicine, Al Jalila Children's Specialty Hospital, Dubai Academic Health Corporation, Dubai, ARE

**Keywords:** cobblestone, forehead swelling, influenza a, pansinusitis, pocus, point of care ultrasound, pott's puffy tumour, sinusitis, streptococcus pyogenes, subperiosteal abscess

## Abstract

Pott's puffy tumor is a subperiosteal abscess that mostly occurs as a rare complication of frontal sinusitis or osteomyelitis of the frontal bone. It can be life-threatening without timely identification and treatment owing to its potential for intracranial extension, causing empyema, meningitis, sinus venous thrombosis, or cerebral abscess. In this case report, we describe the case of a 16-year-old girl who presented with a tender forehead swelling, fever, sore throat, and nasal congestion. A bedside ultrasound scan helped to identify early changes suggestive of frontal sinus infection and abscess formation. Head computed tomography confirmed the diagnosis of Pott's puffy tumor, with pansinusitis as an additional finding. This case highlights early identification of the condition based on clinical presentation and point-of-care ultrasound findings, which enabled us to initiate appropriate intravenous antibiotic therapy in the Emergency Department. Timely initiation of treatment using a multidisciplinary approach led to significant improvement in the clinical outcome for our patient and prevented progression to intracranial extension and other complications.

## Introduction

Pott's puffy tumor (PPT) is a subperiosteal abscess resulting from acute or chronic frontal sinus osteomyelitis, most commonly affecting young adolescents. Patients typically present with upper respiratory tract symptoms and headaches that are accompanied by a tender, circumscribed, doughy, erythematous forehead swelling [1]. The most common imaging modalities used to diagnose PPT are head computed tomography (CT) with contrast and brain magnetic resonance imaging (MRI) with contrast [2]. However, bedside point-of-care ultrasound (POCUS) is emerging as a helpful tool for early identification, although confirmation of the diagnosis can only be achieved using CT or MRI [3,4].

The lesion can often extend intracranially, leading to complications such as meningitis, encephalitis, raised intracranial pressure, or sinus venous thrombosis. Rapid identification, diagnosis, and treatment are crucial to optimizing outcomes [1,2]. Here, we describe a case of PPT diagnosed in a previously healthy 16-year-old female adolescent with a background of sinusitis involving multiple paranasal sinuses. PPT was identified via bedside POCUS and confirmed using head CT.

## Case presentation

A previously healthy 16-year-old girl presented to the Emergency Department (ED) with a seven-day history of fever, dry cough, sore throat, and generalized body aches. She had been diagnosed with influenza B at another medical facility and started on oseltamivir at the onset of these symptoms. Three days before presenting to the ED, the patient noticed a painful swelling that was initially over the right eyelid and then localized to the right forehead. The swelling progressively increased in size and was tender to touch and associated with a headache. She also developed generalized abdominal pain and poor oral intake, with one episode of non-bloody, nonbilious, nonprojectile vomiting. There was no history of trauma or similar past presentation reported.

**Figure 1:**
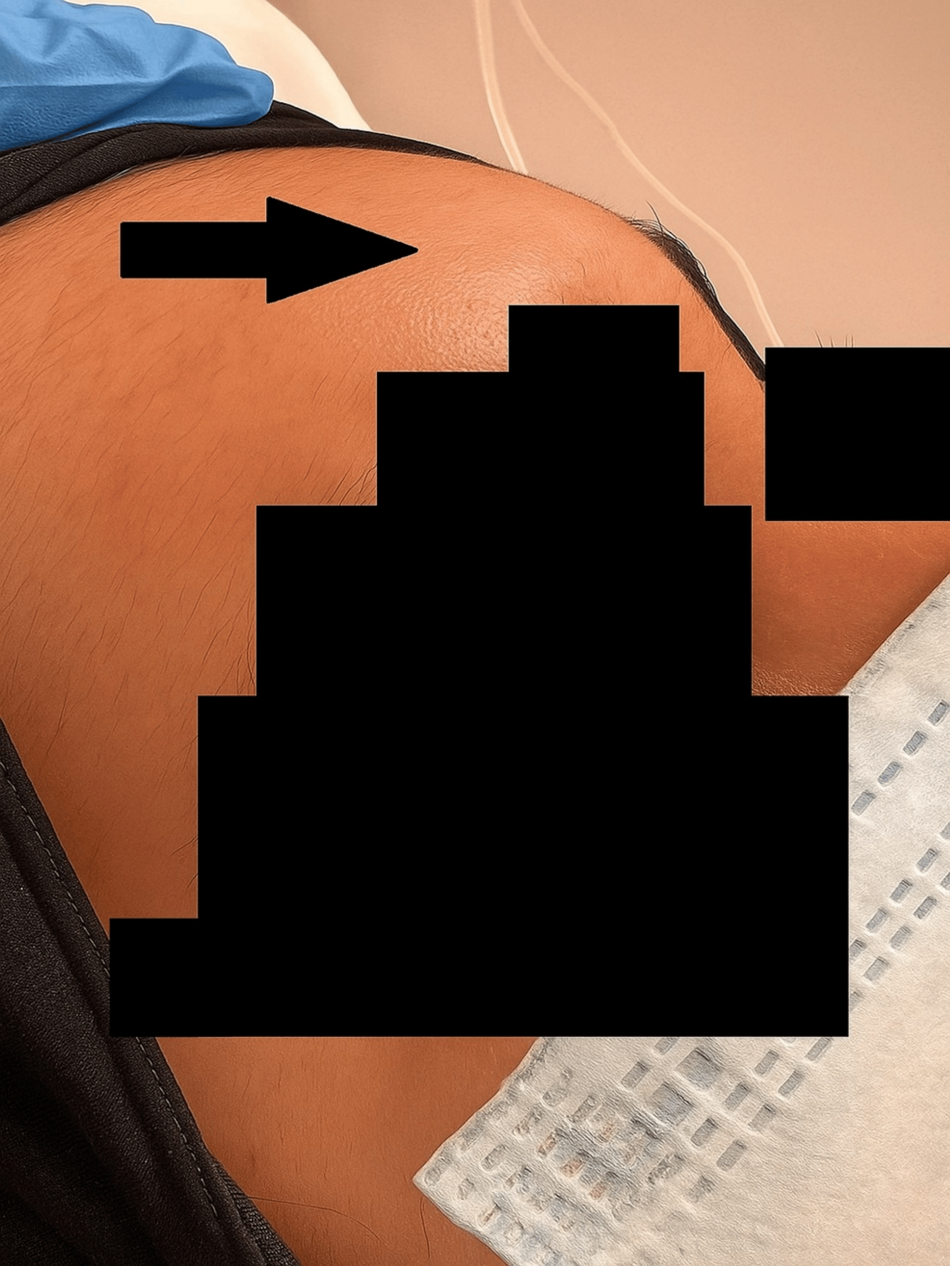
Examination finding of forehead swelling (black arrow).

**Figure 2:**
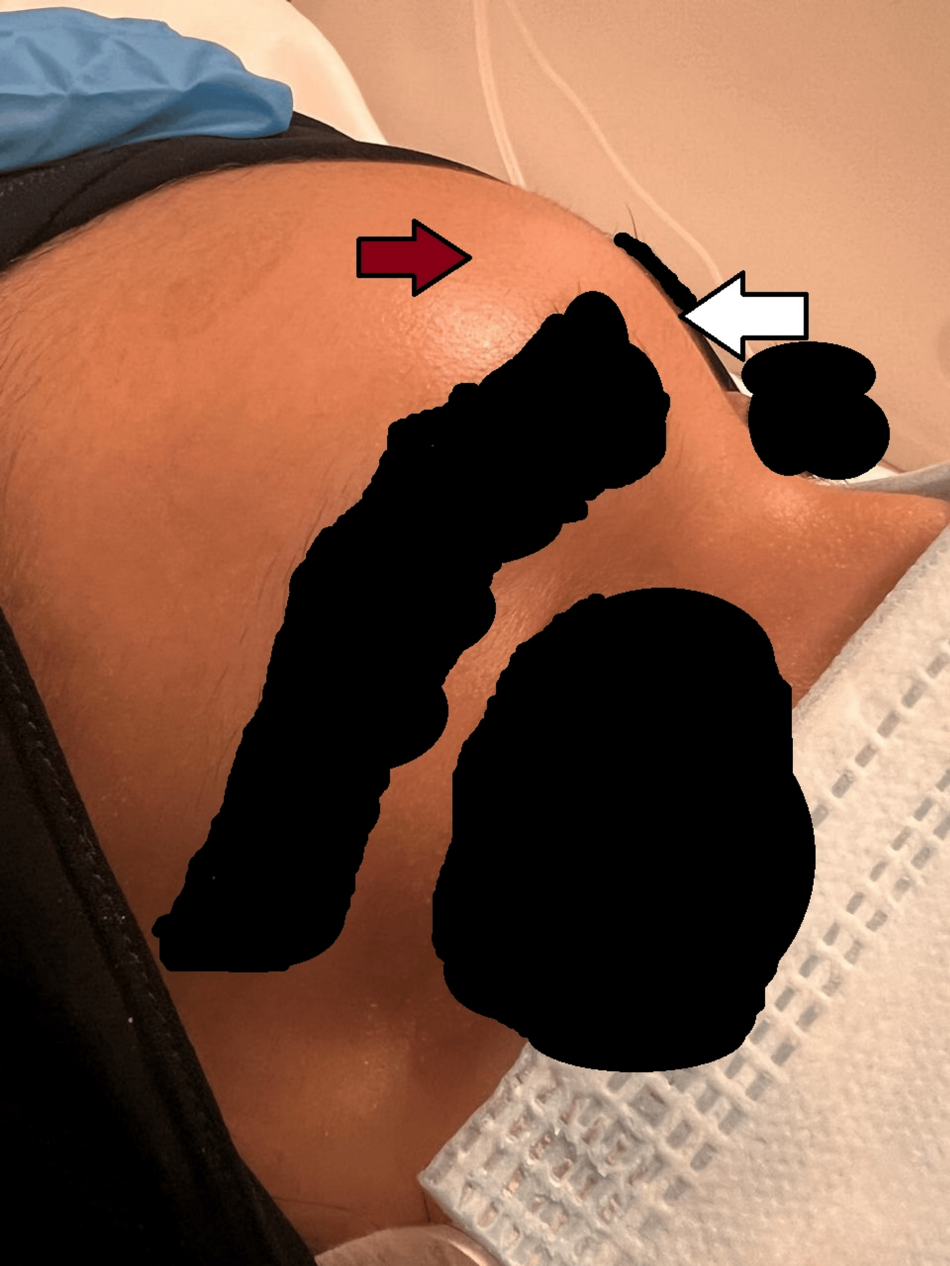
The red arrow highlights the area of the forehead swelling, while the white arrow demonstrates its extension toward the nasal bridge.

On examination, the child was febrile with a low-grade temperature of 37.9°C and tachycardia, with heart rate of 124 bpm (normal range: 60-100 bpm). She was hypotensive, with a blood pressure of 85/56 mmHg and a mean arterial pressure (MAP) of 65 mmHg (normal ranges: systolic 110-131 mmHg, diastolic 64-83 mmHg, MAP: 73-84), findings suggestive of sepsis. Peripheral perfusion was normal, and oxygen saturation was maintained on room air. Her blood pressure normalized following administration of initial fluid bolus after which she was maintained on intravenous fluid. She was conscious, alert, and oriented. There was a single prominent forehead swelling with regular margins that was circular in shape and measured 3 cm × 4 cm in dimensions extending up to the nasal bridge. It was tender on palpation, but there was no overlying erythema or skin discoloration and no bleeding or discharge. Bilateral extraocular movements were unrestricted, and there was no eye redness, eye discharge, or meningeal signs (Figures 1, 2).

**Figure 3:**
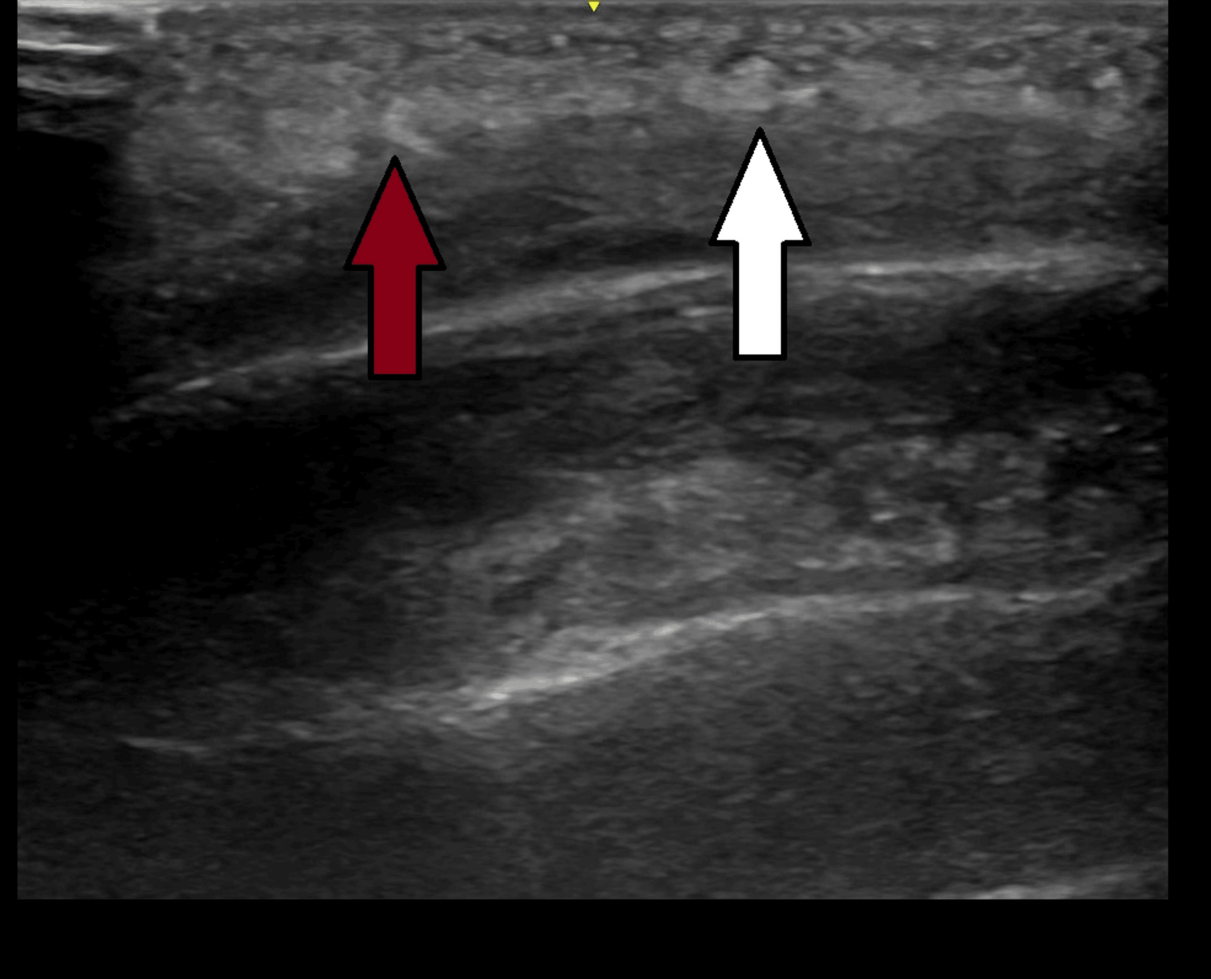
POCUS finding of cobblestone pattern with soft tissue swelling. The red arrow points to the soft tissue swelling, and the white arrow demonstrates a cobblestone pattern observed in the point-of-care ultrasound (POCUS) of the forehead swelling.

**Figure 4:**
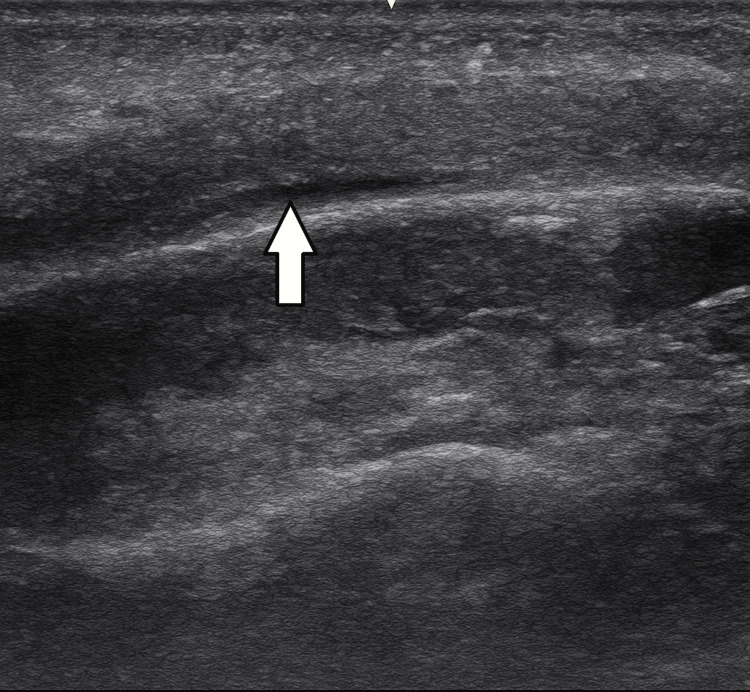
Point-of-care ultrasound showing a thin subperiosteal fluid collection (white arrow).

A bedside point-of-care ultrasound (POCUS) was immediately performed using a high-frequency linear probe over the forehead swelling. The scan revealed soft tissue swelling with a cobblestone appearance and a thin subperiosteal fluid collection (Figures 3, 4). The cobblestone appearance typically reflects edematous changes within the subcutaneous tissue, commonly seen in cellulitis. However, the presence of a subperiosteal fluid collection is indicative of abscess formation, supporting the diagnosis of Pott's puffy tumor.

**Figure 5:**
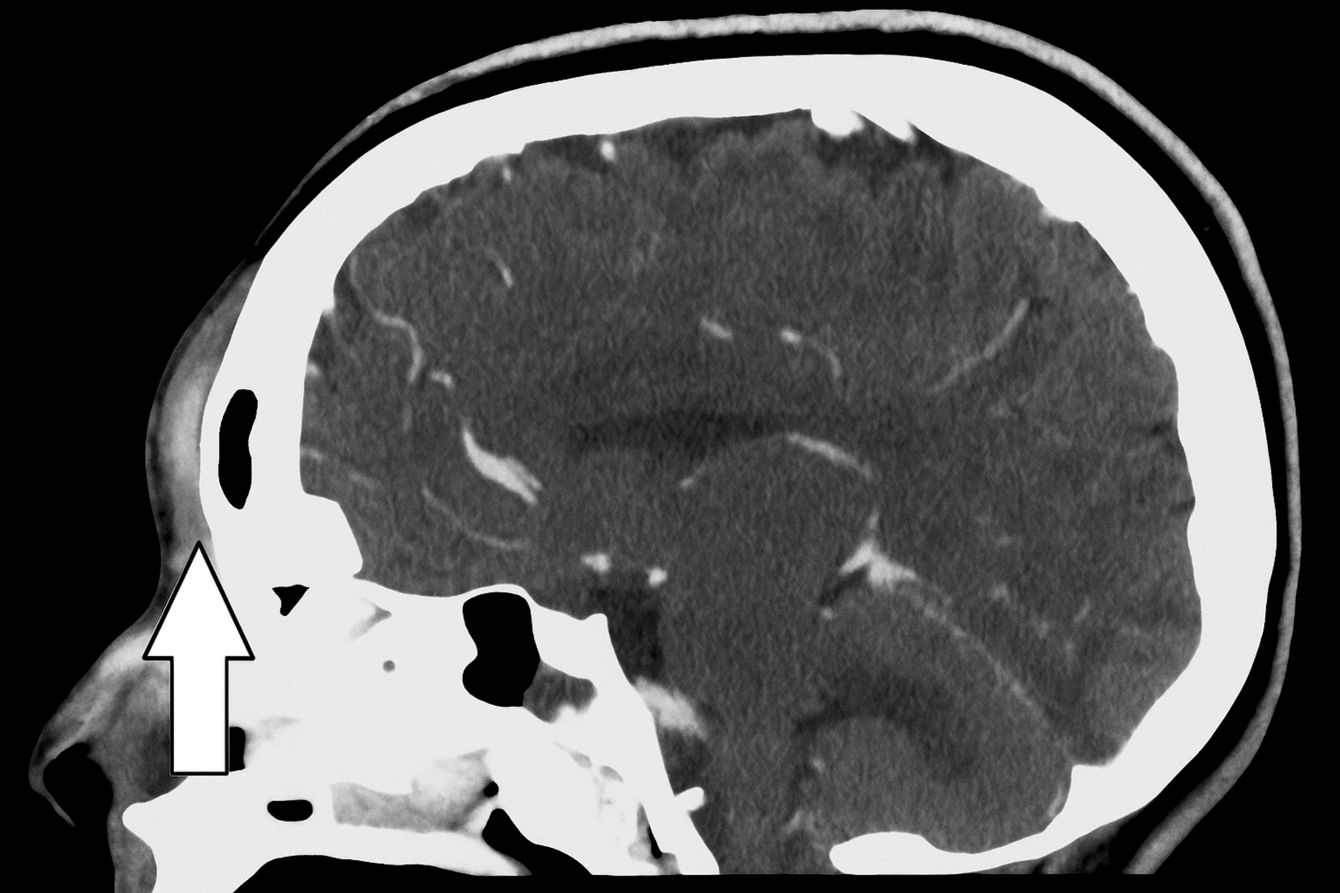
Head CT with contrast showing enhancing soft tissue thickening with a small abscess and air pocket (white arrow) over the anterior frontal region of the skull.

**Figure 6:**
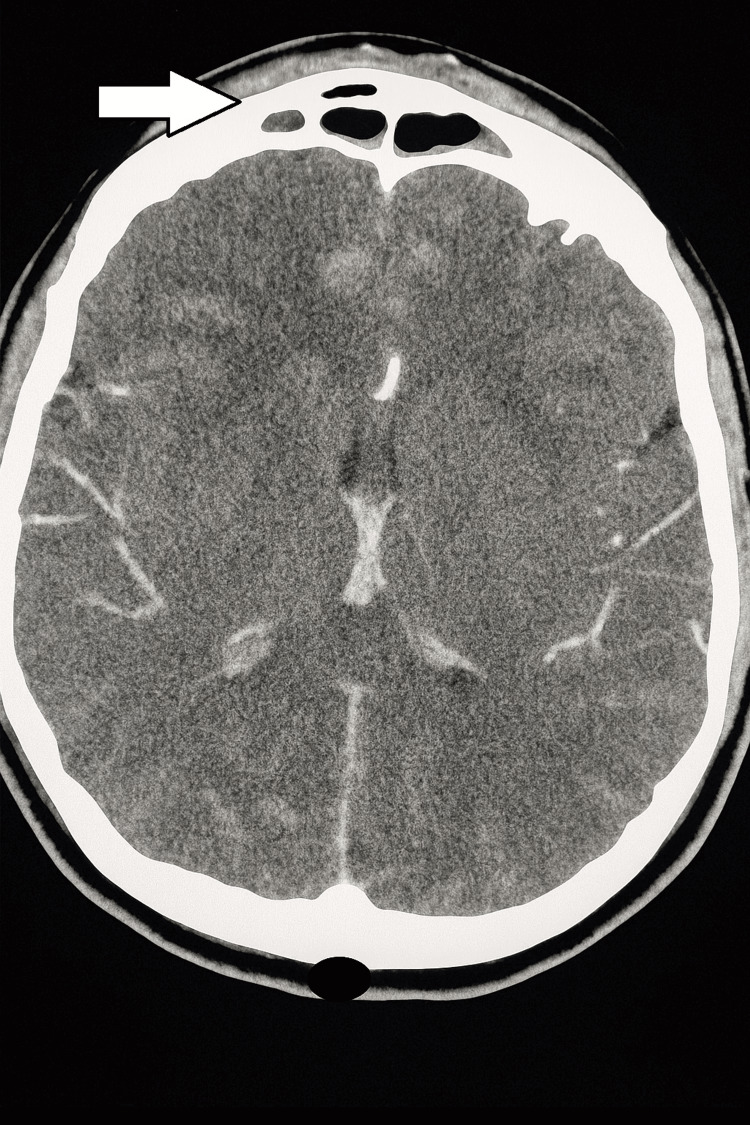
Head CT with contrast showing bony erosion of the frontal sinus with fluid collection within the sinus cavity (white arrow) and no intracranial extension.

A head CT with contrast showed enhancing soft tissue thickening with a small abscess and air pocket over the anterior frontal region of the skull. The dimensions of the abscess were 1.6 cm × 1.5 cm × 0.3 cm. Mucosal thickening was identified in the frontal sinus, bilateral ethmoid sinus, sphenoid sinus, and right maxillary sinus with an increased air-fluid level in the left maxillary sinus. There was no intracranial extension of the anterior frontal lesion (Figures 5, 6).

The imaging findings were consistent with pansinusitis associated with a subperiosteal abscess, confirming PPT. Laboratory investigations showed elevated C-reactive protein 244.8 mg/L (reference range: 0-5 mg/L) and elevated procalcitonin 0.86 ng/mL (reference range: <0.5 ng/mL) with a normal white blood cell count. A respiratory viral nasal panel was positive for influenza B.

Supportive management included intravenous (IV) fluid hydration, antipyretics, and analgesia. Antibiotic coverage was achieved with IV ceftriaxone and IV vancomycin. The patient was admitted to the inpatient unit for further management and observation of possible complications of PPT. Management involved a multidisciplinary approach. An inpatient consultation with pediatric ENT yielded a recommendation to continue IV antibiotics and add nasal wash, xylometazoline nasal drops, and mometasone nasal spray.

Blood culture collected at the time of hospital admission and a skin swab taken from the PPT site grew *Streptococcus pyogenes*, which was found to be sensitive to the administered antibiotics. The Pediatric Infectious Disease team recommended continuing IV ceftriaxone to cover the streptococcal infection and discontinuing IV vancomycin. A repeat blood culture obtained 48 hours after the initial positive culture confirmed clearance of the infection.

With this management plan, significant clinical improvement occurred, including an improving fever curve, resolution of headache, and reduction in the size of the forehead swelling. Inflammatory markers repeated 48 hours after initiating management showed a significant downward trend, eliminating the need for surgical intervention.

On day 6 of admission, antibiotic coverage was optimized from IV ceftriaxone to IV ampicillin as recommended by the Infectious Disease team, with a plan to complete a 10-day course of IV antibiotics as an inpatient followed by eight weeks of oral amoxicillin treatment as an outpatient. In view of significant clinical improvement, negative repeat blood culture, and down-trending inflammatory markers after completing the 10-day course of IV antibiotics, the patient was discharged on oral amoxicillin and mometasone nasal spray with planned follow-ups in the ENT and infectious disease clinics.

## Discussion

PPT is a subperiosteal abscess resulting from associated frontal skull osteomyelitis. Several case reports have described this clinical condition and its varied presentations. Most often, it affects young adolescents, typically as a complication of frontal sinusitis.

The second most common cause of this condition is head trauma to the frontal area caused by direct extension of wound infection or contamination of the frontal bone. Infrequent causes include cranial/frontal surgery, dental infection, wrestling, cocaine abuse, and insect bites [1,2]. Owing to a peak in vascular development in the diploic circulation around puberty and the growth of the frontal sinus, PPT most commonly affects adolescents [2]. Our patient, a 16-year-old girl, belonged to this age group.

Clinically, PPT is characterized by a circumscribed, tender, “doughy” erythematous forehead swelling associated with fever, headache, nasal discharge, or increased intracranial pressure. Other signs and symptoms may include periorbital swelling, nausea/vomiting, cutaneous fistulas, meningitis, or encephalitis [1,2]. On examination, our patient presented with similar findings.

The gold standard imaging modalities to confirm the diagnosis of PPT are head CT with contrast and brain MRI with contrast. On a CT scan, sinusitis and signs of osteomyelitis, such as bone opacification and destruction, along with potential involvement of the frontal sinuses and air-trapping in bone or soft tissue, can be observed [1,3,4]. In the case described here, a CT scan clearly identified soft tissue enhancement with fluid collection in the frontal sinus, as well as signs of sinusitis involving multiple sinuses (frontal, ethmoidal, sphenoid, and maxillary sinuses).

MRI with gadolinium enhancement is superior for soft tissue presentation and is therefore the gold standard in evaluating suspected intracranial complications of PPT. This imaging modality should always be performed if neurological symptoms are observed. MRI is also the study of choice for follow-up during the postoperative period, as it eliminates radiation exposure [1,3,4]. Bone scintigraphy has also been described, although it is not often used in the modern setting. It is more sensitive than CT for early osteomyelitis but has limited use if clinical signs are present [1].

Another emerging imaging modality for evaluating these cases is bedside POCUS. In the pediatric emergency, this modality can prove advantageous in comparison to CT and MRI owing to the absence of ionizing radiation, no need for anesthesia, and greater accessibility to equipment. Therefore, POCUS offers a more accessible and less risky imaging option in the pediatric age group [5].

On ultrasound of the forehead swelling, discontinuity of the frontal bone and a hypoechoic subcutaneous mass consistent with a subperiosteal abscess can commonly be identified [3,4]. In our case, bedside POCUS clearly demonstrated a cobblestone pattern of the forehead swelling with thin subperiosteal fluid collection, which has been described as a sign of PPT [3,4].

Once PPT is identified, a multidisciplinary care team approach is highly recommended for appropriate treatment. This approach may include the involvement of General Pediatrics, Otolaryngology, Neurosurgery, Infectious Disease, and Ophthalmology teams, depending on the patient's clinical presentation and extension of the subperiosteal abscess [6].

Most cases of PPT occur against a background of upper respiratory tract infections that are polymicrobial in nature. Thus, various types of streptococci, staphylococci, and anaerobes are the most common organisms causing PPT. Broad-spectrum antibiotic coverage is most suitable for empiric treatment of this condition [2]. It is essential to choose antibiotics with adequate blood-brain barrier penetration for intracranial coverage. Options include penicillin or vancomycin, third-generation cephalosporins, and metronidazole. Once culture results are available, broad-spectrum antibiotics can be changed to more targeted therapy [1,2,6].

Patients with anatomical variations causing PPT require surgical intervention. Surgical options include craniotomy, trephination, frontal sinus obliteration, and minimally invasive endoscopic frontal sinusotomy [1,3]. Poor prognosis is usually associated with delayed onset of treatment and the presence of intracranial complications such as meningitis, encephalitis, epidural abscess, or raised intracranial pressure [1].

## Conclusions

PPT is a rare condition that is most commonly seen in the pediatric and adolescent age groups, and its complications pose a significant risk owing to potential intracranial extension. Such risk makes early diagnosis and treatment crucial for improving clinical outcomes. While CT and MRI with contrast are commonly used for diagnosis, bedside ultrasound imaging is becoming an emerging tool for identifying such conditions in pediatric patients. PPT can be initially identified using bedside ultrasound, with confirmation achieved through CT or MRI.

The case presented here emphasizes the role of bedside POCUS, which is a low-risk tool that involves no radiation. It can be safely used in the pediatric age group and is easily accessible in any emergency setting. It is reliable in the early identification and diagnosis of PPT, reducing time to treatment and thereby drastically improving prognosis and preventing life-threatening complications.
